# Solution to the SLAM Problem in Low Dynamic Environments Using a Pose Graph and an RGB-D Sensor

**DOI:** 10.3390/s140712467

**Published:** 2014-07-11

**Authors:** Donghwa Lee, Hyun Myung

**Affiliations:** Urban Robotics Laboratory, Korea Advanced Institute of Science and Technology (KAIST), 291 Daehak-ro, Yuseong-gu, Daejeon 305-701, Korea; E-Mail: leedonghwa@kaist.ac.kr

**Keywords:** simultaneous localization and mapping (SLAM), low dynamic environment, pose graph, RGB-D (red-green-blue depth)

## Abstract

In this study, we propose a solution to the simultaneous localization and mapping (SLAM) problem in low dynamic environments by using a pose graph and an RGB-D (red-green-blue depth) sensor. The low dynamic environments refer to situations in which the positions of objects change over long intervals. Therefore, in the low dynamic environments, robots have difficulty recognizing the repositioning of objects unlike in highly dynamic environments in which relatively fast-moving objects can be detected using a variety of moving object detection algorithms. The changes in the environments then cause groups of false loop closing when the same moved objects are observed for a while, which means that conventional SLAM algorithms produce incorrect results. To address this problem, we propose a novel SLAM method that handles low dynamic environments. The proposed method uses a pose graph structure and an RGB-D sensor. First, to prune the falsely grouped constraints efficiently, nodes of the graph, that represent robot poses, are grouped according to the grouping rules with noise covariances. Next, false constraints of the pose graph are pruned according to an error metric based on the grouped nodes. The pose graph structure is reoptimized after eliminating the false information, and the corrected localization and mapping results are obtained. The performance of the method was validated in real experiments using a mobile robot system.

## 1. Introduction

Simultaneous localization and mapping (SLAM) is a key problem for the robotics community [1–11]. Originally, it was assumed that the SLAM technique can only be performed in static environments. This assumption remains valid for the verification and comparison of a variety of SLAM algorithms but the real world is a dynamic environment. In recent years, SLAM has been developed for use in dynamic environments [7–11], but many of these methods rely on expensive laser range finder (LRF) sensors. Nevertheless, in highly dynamic environments, since vision sensors can readily detect the moving object, visual SLAM delivers good performance [11]. However, if the object positions change over long intervals, it is difficult to recognize these movements using vision sensors alone. This problem was defined in [8] (where they used an LRF sensor) and referred to as a low dynamic environment.

In the present study, we propose a novel SLAM method for low dynamic environments, which is based on an RGB-D (red-green-blue depth) sensor. RGB-D sensors generate a colored two-dimensional (2D) image and depth data concurrently [12], which allows the constraints between other places to be obtained easily. These sensors are also relatively cheap (less than $ 300) compared with LRF sensors. The proposed method is optimized by using a pose graph structure [4–6], which stores the full trajectory information and sensor measurements as constraints. In the pose graph, the dynamic objects cause false constraints. Furthermore, the false constraints form a group when the moved objects are observed for a while. Therefore, to remove falsely grouped constraints efficiently, the proposed method first groups nodes that represent robot poses of the trajectory according to the grouping rules with noise covariances. Next, the false constraints generated by the dynamic objects are pruned according to an error metric that is based on the grouped nodes. The pose graph structure is reoptimized after eliminating the false information, and the corrected robot trajectory and a three-dimensional (3D) point cloud map are obtained.

Recently, max-mixture, vertigo, and dynamic covariance scaling (DCS)-based graph SLAM algorithms have been introduced to remove false constraints from the graph SLAM [13–15]. However, these constraints are focused on false constraints generated by coincidence, whereas our situation is an inevitable consequence of dynamic environments. Therefore, these types of algorithms are not suitable for eliminating the grouped false constraints generated by moving objects. More details of the algorithms and results of applying the algorithms in a low dynamic environment will be provided in Section 3.2.

The main contribution of this paper is in using a relatively cheap sensor and providing an effective error metric with the pose graph to overcome the low dynamic environments. Unlike earlier studies that use expensive sensors, the proposed method uses a relatively cheap RGB-D vision sensor. Then, using only the pose graph information, false constraints from the low dynamic situations can be detected effectively with an error metric and node grouping rules. After that, the false informations can be removed easily, and then the corrected robot trajectory and 3D map are finally obtained.

The remainder of this paper is organized as follows. The second section provides a review of pose graph SLAM and the RGB-D SLAM system. The third section describes the proposed SLAM method for low dynamic environments. The results of the actual experiments are presented in the fourth section. The final section offers some concluding remarks.

## 2. Pose Graph-Based RGB-D SLAM

### 2.1. Pose Graph SLAM

**Figure 1.**
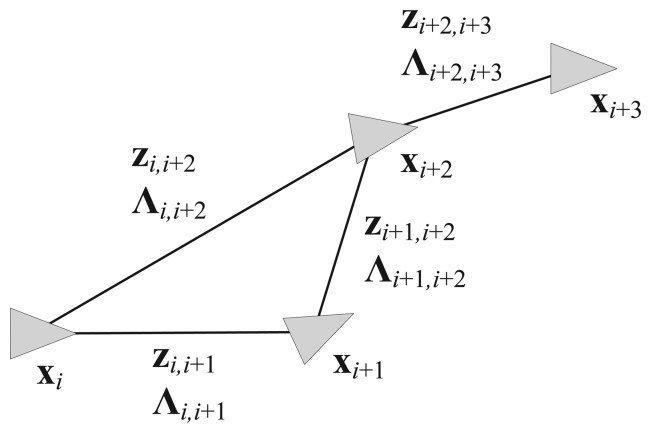
Graphical model of pose graph SLAM.

Our proposed method is based on pose graph SLAM [4–6]. Graph SLAM basically comprises nodes and edges. The nodes represent robot poses or landmark positions in the map, while the edges constrain the nodes based on the relative measurements between pairs of nodes. In pose graph-based SLAM, the robot poses are used only as the nodes and a pair of nodes connected to the same landmark acquire a new edge after removing the landmark node. Figure 1 shows a graphical model of pose graph SLAM. The pose graph SLAM structure is useful in situations where it is difficult to define an exact landmark such as LRF-based SLAM. There are also some advantages in terms of the computational speed or the transformation of the graph structure because pose graph SLAM has a more compact information structure than general graph SLAM.

The pose graph SLAM algorithm optimizes the full trajectory of a robot using the maximum-likelihood estimation (MLE) method, as follows:

(1) $${\text{x}}^{*}=\arg\underset{\text{x}}{\min}\frac{1}{2}\underset{\langle i,j\rangle \in \text{S}}{\text{∑}}{\text{r}}_{i,j}^{T}(\text{x})\Lambda_{i,j}{\text{r}}_{i,j}(\text{x})$$

where **x** is the robot pose vector with a full trajectory, **r***_i,j_* is the residual of the predicted and observed relative poses between the *i*-th and *j*-th nodes, Λ*_i,j_* denotes the measurement information matrix, and 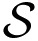 represents the set of edges that connects the nodes. The residual **r***_i,j_* is represented as

(2) $${\text{r}}_{i,j}(\text{x})={\text{h}}_{i,j}(\text{x})-{\text{z}}_{i,j}$$

where **h***_i,j_* (**x**) represents the prediction model for two nodes and **z***_i,j_* is the measurement value obtained from sensors. Therefore, the optimization of the robot trajectory denotes the minimization of Mahalanobis distance [16] of the residuals. Since the residual **r***_i,j_* is generally a nonlinear function, the pose graph SLAM is a nonlinear least square problem and the solution is obtained iteratively by

(3) x←x+Δx

where Δ**x** is the solution of the following problem:

(4) HΔx=−g

where **H** and **g** are represented by Equations (5) and (6), respectively, and **J***_i,j_* is Jacobian of the residual **r***_i,j_* with respect to **x**, which is obtained using Equation (7).

(5) $$\text{H}=\underset{\langle i,j\rangle \in \text{S}}{\text{∑}}{\text{J}}_{i,j}^{T}\Lambda_{i,j}{\text{J}}_{i,j}$$

(6) $$\text{g}=\underset{\langle i,j\rangle \in \text{S}}{\text{∑}}{\text{J}}_{i,j}^{T}\Lambda_{i,j}{\text{r}}_{i,j}(\text{x})$$

(7) $${\text{J}}_{i,j}={\frac{\partial {\text{r}}_{i,j}\text{(}\overset{⌣}{\text{x}}\text{)}}{\partial \overset{⌣}{\text{x}}}|}_{\overset{⌣}{\text{x}}=\text{x}}$$

Recently, a variety of graph SLAM algorithms, such as TORO, g2o, and iSAM [4–6], has been developed to improve the computational efficiency of this process. In the present study, the iSAM algorithm is used to optimize the robot's full trajectory. iSAM reduces the computational time considerably based on sparse linear algebra [6].

### 2.2. RGB-D SLAM System

**Figure 2.**
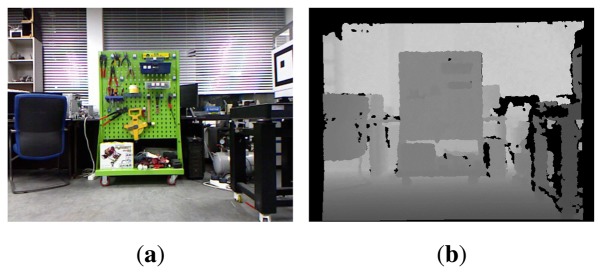
RGB-D sensor data. (**a**) RGB 2D image; (**b**) Per-pixel depth data.

**Figure 3.**
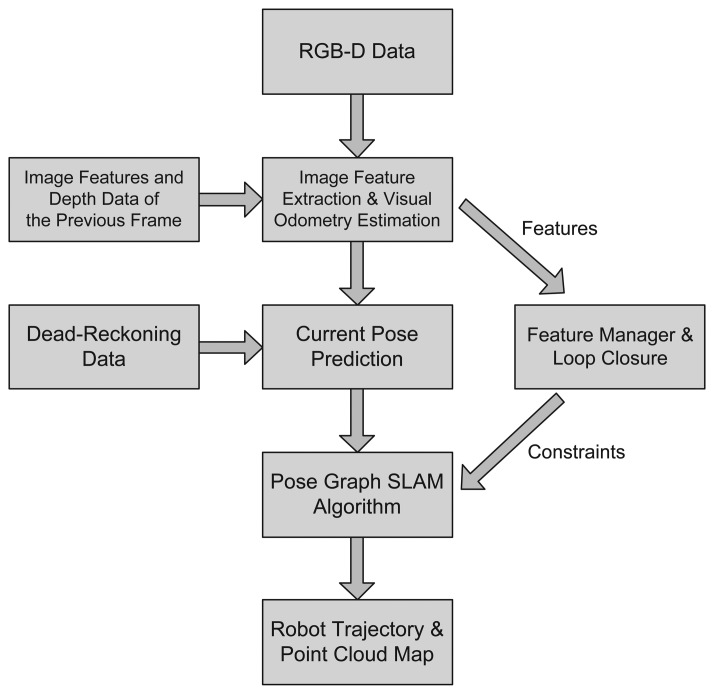
Processing steps required by the RGB-D SLAM system.

In the present study, the proposed SLAM method is implemented and validated using an RGB-D SLAM system. RGB-D sensors such as Microsoft Kinect provide depth information as well as color information [12]. Figure 2a and b shows a color image and the per-pixel depth data obtained from an RGB-D sensor. The RGB-D SLAM system utilizes the RGB 2D image and depth data from an RGB-D sensor and robot's dead-reckoning data. The processing steps required by the system are illustrated in Figure 3. First, the 2D image features are extracted using feature extraction algorithms such as the scale-invariant feature transform (SIFT) [17] and speeded-up robust features (SURF) [18]. Each feature can be located at a point in the 3D coordinate space using the depth data and the focal length information from the camera sensor. These features are used for visual odometry estimation based on comparisons between the current and preceding frames using feature matching and a RANSAC (RANdom SAmple Consensus) algorithm [19]. Next, the robot's dead-reckoning data is fused with the visual odometry estimate to predict the current robot pose. This prediction constrains the previous and current nodes. A feature manager gathers the overall features from the image frames and, based on comparison between the current and previous features, the current node is matched to the past nodes of the graph. This matching procedure is called loop closure detection. The robot pose prediction and loop closure measurement set the constraints between the graph nodes and the full trajectory of the robot is formed as a pose graph structure. After optimizing the pose graph using the pose graph SLAM algorithm mentioned in the previous section, the corrected robot trajectory and 3D map can be obtained. The steps are all performed in real-time. A detailed explanation of this system is given in [20,21].

## 3. Proposed SLAM method

### 3.1. SLAM in Low Dynamic Environments

**Figure 4.**
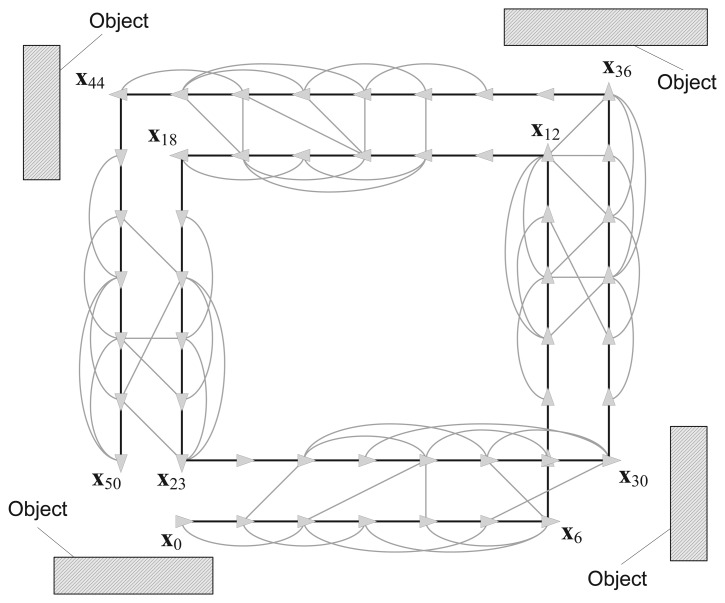
Example of pose-graph SLAM in a static environment.

Until recently, it was assumed that most SLAM algorithms can only be performed in static environments. Figure 4 shows an example of 2D pose graph SLAM in these environments. This example is based on the RGB-D SLAM system discussed in the previous section. It is assumed that the robot is equipped with the RGB-D sensor that points ahead to gather vision and depth data as it moves. The robot starts at the first node **x**_0_ and moves along double-rectangular paths. The nodes are connected by edges, which are generated using dead-reckoning estimation and by an image feature-matching technique. In this case, the image features are extracted from four surrounding objects and the nodes that observe the same object are connected to each other based on common image features. The black bold edges connecting two nodes in Figure 4, which are located immediately next to each other, represent the pose prediction results. The pose prediction is obtained from the visual odometry estimation based on a comparison with the previous RGB-D data and the dead-reckoning of the robot. We refer to these edges as *prediction edges*. The gray thin edges are produced by loop closure detection using the gathered features. These edges are referred to as *measurement edges*.

**Figure 5.**
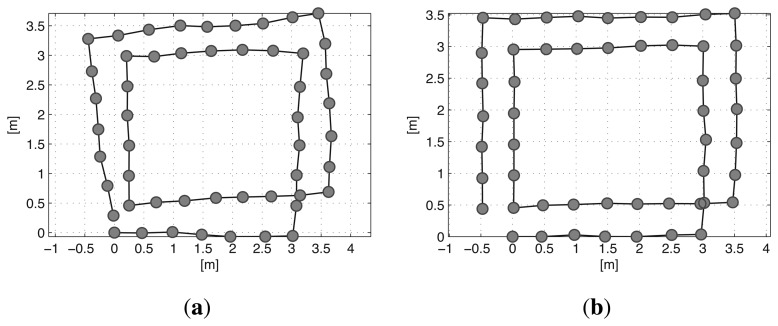
Full trajectory estimation; (**a**) Using only the prediction results. (**b**) Optimized using the graph SLAM algorithm.

Every edge has prediction and measurement uncertainties, which are represented by Gaussian noise with information matrices Λ*_i,j_*. Figure 5a shows the full trajectory of the robot estimated using prediction edges only. The path of the robot is distorted by the noise of the edges. In Figure 5b, the robot trajectory is optimized by the graph SLAM algorithm using all the prediction and measurement edges of the graph, and a corrected rectangular path is obtained.

In contrast to the static situation, objects can move or be moved in dynamic environments. Examples of this type of object movement include people, vehicles, and furniture. The moving objects create false constraints between the nodes and general SLAM algorithms might fail to optimize graphs. However, relatively fast-moving objects such as people and cars can be detected frame by frame using a variety of moving object detection algorithms. By contrast, very low dynamic objects, such as chairs, tables, sofas, and doors are difficult to detect because the movement occurs over relatively long intervals. These situations are referred to as low dynamic environments in [8]. In other words, in this study, the dynamic environments are classified into highly dynamic and low dynamic environments. The low dynamic environments are defined as situations where the movements occur between different visitations. Thus, the typical sensors cannot detect the movements frame by frame using a variety of conventional moving object detection algorithms. Therefore, every environment that meets the above conditions can be referred to as the low dynamic environment regardless of the time scale. The environments that are not included to the low dynamic environments are defined as the highly dynamic environments.

**Figure 6.**
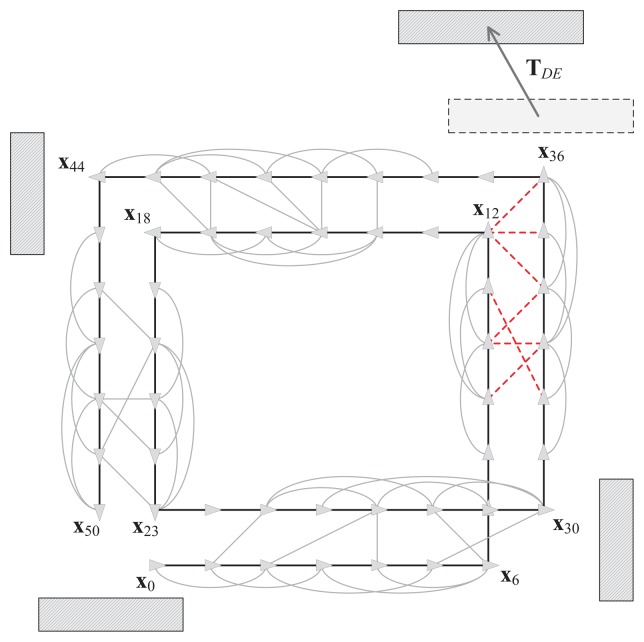
Example of pose graph SLAM in a low dynamic environment.

Figure 6 shows an example of a low dynamic environment.

During the first visit to the object in the top left corner (**x**_6_ to **x**_12_), the objects are placed in the same position as that shown in Figure 4.

**Figure 7.**
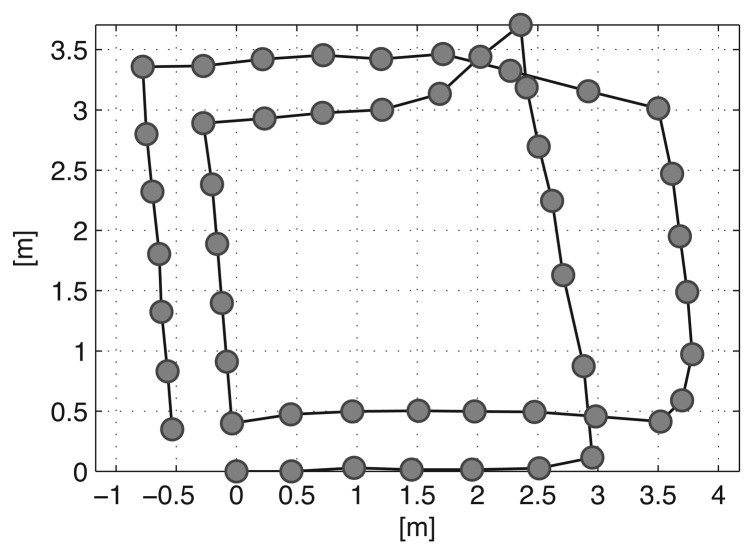
Distorted trajectory by the conventional graph SLAM algorithm due to a low dynamic object.

Before the second visit (**x**_30_ to **x**_36_), the object moves according to the transformation matrix **T***_DE_*. The relocation of the object affects the constraints (red and dotted edges) between the two visits and the result of the pose graph optimization is distorted, as shown in Figure 7. Large errors are induced in the overall trajectory because of the severe distortion in the right part of the graph. Thus, the false constraints need to be removed to avoid incorrect SLAM results. Furthermore, the false constraints form a group because they see the moved object for a while. Therefore, before removing the constraints, a grouping constraints procedure will increase efficiency of a constraint pruning algorithm in these situations.

In the next section, we will apply previous constraints pruning algorithms to the low dynamic environments. After that, to solve this problem in an efficient manner, we will propose a grouping method and a constraint pruning algorithm in the following sections.

### 3.2. Previous Constraints Pruning Approaches

**Figure 8.**
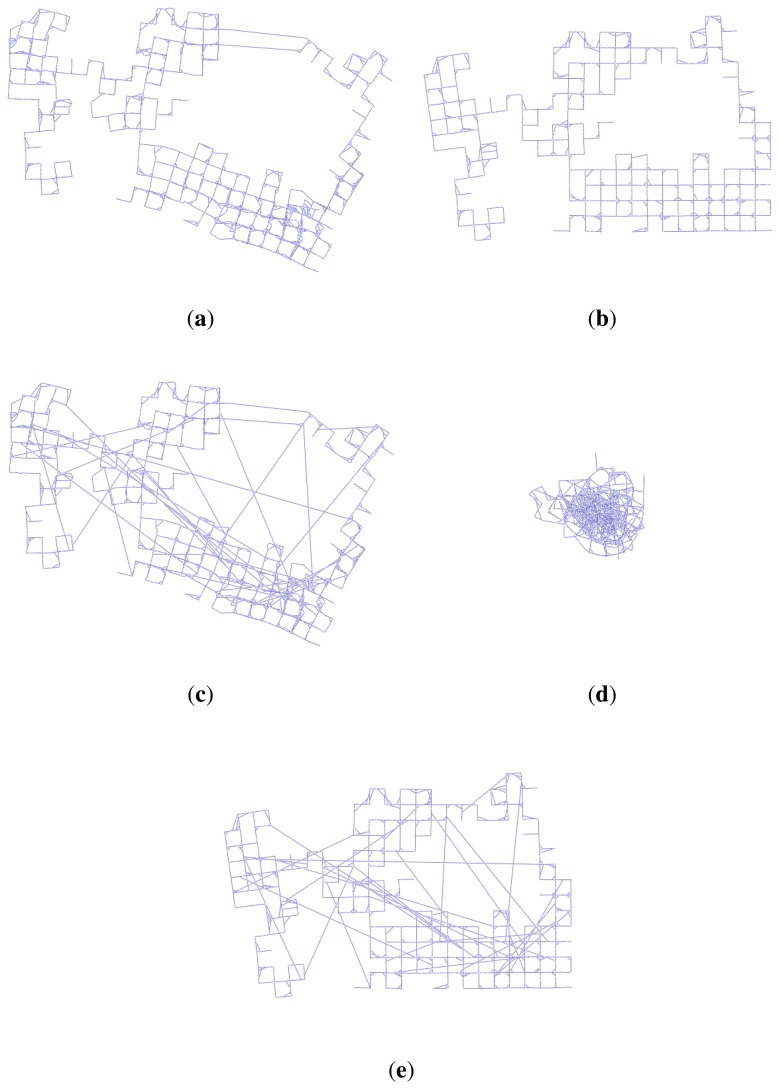
Example of the graph optimization with false constraint edges for the synthetic Manhattan world dataset [22]. 30 false constraints are added to the graph for evaluating different SLAM algorithms. (**a**) Before optimizing the graph with no false constraints; (**b**) Optimizing the graph with false constraints using the conventional graph SLAM; (**c**) Before optimizing the graph with false constraints; (**d**) Optimizing the graph with false constraints using the conventional graph SLAM; (**e**) Optimizing the graph with false constraints using the dynamic covariance scaling (DCS)-based graph SLAM [15].

To remove false constraint edges of the graph SLAM, several algorithms, such as max-mixture, vertigo, and dynamic covariance scaling (DCS)-based graph SLAM algorithms, have been suggested [13–15]. These algorithms select false constraints using multiple-hypothesis methods or switchable selection factors during a least square optimization process. For proper working of these algorithms, the false constraints should have been generated by coincidence, so that each of them has a non-causal relationship to the others. Figure 8 shows an example of the graph optimization with false constraint edges for the synthetic Manhattan world dataset [22]. Figure 8a represents raw data without any false constraints, and their optimization result using a conventional graph SLAM algorithm is shown in Figure 8b. Next, randomly generated 30 false constraints are added to the dataset (Figure 8c), and the optimization result using conventional graph SLAM is shown in Figure 8d. Due to the false constraints, the conventional graph SLAM gives a severely distorted map. Figure 8e shows the result of applying the DCS-based graph SLAM algorithm [15]. The algorithm detects false constraints and optimizes the graph correctly, which shows the same result with Figure 8b. The other pruning methods also give the same results using the DCS-based algorithm.

**Figure 9.**
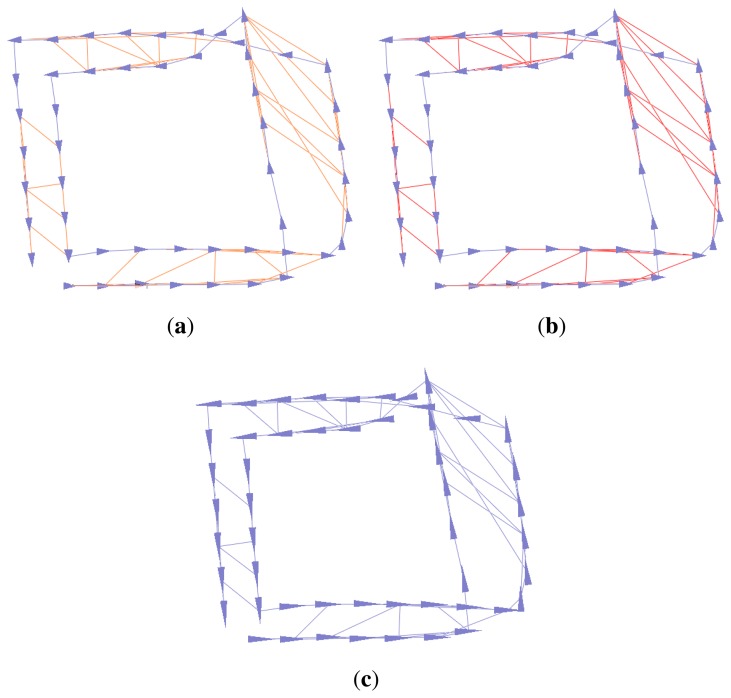
Results of applying different SLAM algorithms for a low dynamic environment. (**a**) Max-mixture algorithm [13]; (**b**) Vertigo algorithm [14]; (**c**) Dynamic covariance scaling (DCS)-based algorithm [15].

To find out the usefulness of the previous methods in our situation, we apply the three algorithms to the low dynamic environment, and their results are shown in Figure 9. The algorithms do not prune any false constraints in this environment, and the maps are still distorted. The false constraints generated by moving objects are inevitable consequence of the low dynamic environment. Furthermore, they appear for a while during their observation of the same moved object, resulting in the formation of a group of false constraints. These grouped constraints are hard to be removed by the previous algorithms because they are effective when the constraints are formed by coincidence. Therefore, to solve this problem, we propose an efficient algorithm in the following sections.

### 3.3. Grouping Nodes

In this part, we propose a method for grouping nodes to facilitate the efficient pruning of constraints. The method employs a covariance merging scheme and two rules for selecting a sequence of nodes. Figure 6 shows the necessity of the node grouping method. Several false constraints emerge from the object movement because the robot has a vision sensor that points ahead and it observes the same object for a period. In this case, if two groups related to the moving object (the first and the second visiting groups to the low dynamic object) can be formed, the false constraints are established between the two visiting groups. After that, if it is revealed that the relationship between the two groups is incorrect, all of the constraints that connect the groups can be pruned concurrently.

**Figure 10.**
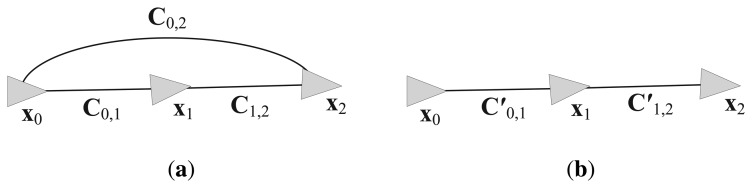
Example showing the merging of the covariances of edges. (**a**) Before merging **C**_0,2_ to **C**_0,1_ and **C**_1,2_; (**b**) After merging, **C**′_0,1_ and **C**′_1,2_ are updated.

The covariance merging process for grouping is described in the followings. The uncertainty score of each prediction edge is calculated to group the nodes. All of the edges have information matrices Λ*_i,j_* between the *i*-th and *j*-th nodes. The information matrix is the inverse of the measurement covariance matrix **C***_i,j_*. The probabilistic distribution of the measurement edges can be merged to the prediction edges using the parallel summation rules of Gaussian distributions [23]. Figure 10 shows an example of merging the covariances of edges. The covariance **C**_0,2_ is merged to the prediction edges **C**_0,1_ and **C**_1,2_, and the updated covariances **C**′_0,1_ and **C**′_1,2_ are obtained as

(8) $${{\text{C}}^{\prime }}_{0,2}={\left[{\left({\text{C}}_{0,1}+{\text{C}}_{1,2}\right)}^{-1}+{\text{C}}_{0,2}^{-1}\right]}^{-1}$$

(9) $${{\text{C}}^{\prime }}_{0,1}={{\text{C}}^{\prime }}_{0,2}\frac{{\text{C}}_{0,1}}{{\text{C}}_{0,1}+{\text{C}}_{1,2}}$$

(10) $${{\text{C}}^{\prime }}_{1,2}={{\text{C}}^{\prime }}_{0,2}\frac{{\text{C}}_{1,2}}{{\text{C}}_{0,1}+{\text{C}}_{1,2}}$$

In the present study, we assume that the robot moves on a 2D plane, so the node **x** and the covariance matrix **C** are represented as

(11) $$\text{x}={\left[\begin{array}{ccc} x & y & \theta \end{array}\right]}^{T}$$

(12) $$\text{C}=\left[\begin{array}{l} \begin{array}{ccc} \sigma_{x}^{2} & \sigma_{xy}^{2} & \sigma_{x\theta }^{2} \end{array}\\ \begin{array}{ccc} \sigma_{xy}^{2} & \sigma_{y}^{2} & \sigma_{y\theta }^{2} \end{array}\\ \begin{array}{ccc} \sigma_{x\theta }^{2} & \sigma_{y\theta }^{2} & \sigma_{\theta }^{2} \end{array} \end{array}\right]$$

To apply the covariance merging scheme to our SLAM system, two rules are defined as follows:

1. The measurement edges subject to a certain condition are merged to the prediction edges. The condition is that all nodes between two nodes connected by the measurement edge have to be within a certain angle bound. In other words, the bearing differences between all nodes cannot exceed a certain angle. For example, as shown in Figure 6, the nodes between **x**_34_ and **x**_36_ are within an angle bound of 20°, thus the measurement edge that connects x_34_ to x_36_ is subject to the condition. However, the edge that connects **x**_12_ to **x**_36_ does not meet this requirement because the bearing differences of some nodes between **x**_12_ and **x**_36_ exceed 20°.
2. Given the condition described above, the prediction edges are merged with the measurement edges within the angle bound described above. In this situation, it is assumed that the robot heading is the *x* axis of the robot-fixed coordinate system, which means that the covariances of the prediction edges are affected mostly by *σ_x_*. Therefore, the covariance matrix can be approximated to a single variable as $\text{C}\approx \sigma_{x}^{2}$, and the parallel summation of the covariance matrices becomes simple algebra.

**Figure 11.**
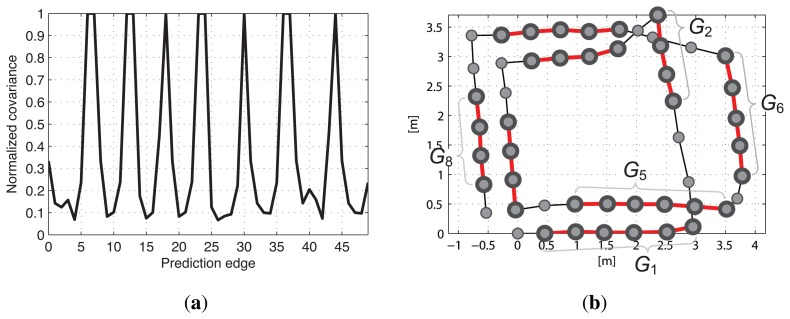
(**a**) Normalized covariance values of the prediction edges; (**b**) Result obtained after grouping the nodes. The red and bold lines represent the grouped nodes. In this case, eight groups are formed, *G*_1_ to *G*_8_.

Figure 11a represents the covariance values of the prediction edges merged according to the two rules, which have been normalized by the maximum value. The parallel summation of the Gaussian distributions reduces the covariance value, which also means that the uncertainty of the prediction edge to which several measurement edges belong is decreased. The nodes with uncertainty values less than 0.3 are grouped and eight groups, *G*_1_ to *G*_8_, are formed as shown in Figure 11b (red and bold lines), where *G_k_* represents the *k*-th node group and *k* is numbered sequentially according to the time. A number of edges exist in the same group nodes that see the common object, thus the results obtained after grouping the nodes facilitate the effective removal of the false constraints.

### 3.4. Pruning Constraints

Next, we propose an error metric that uses the grouped nodes to find the false constraints in an efficient manner. The error value *E_k,l_* is defined as the average Mahalanobis distance [16] of the edges that connect the *k*-th and *l*-th node groups as

(13) $$E_{k,l}=\frac{1}{N_{k,l}}\underset{\langle i,j\rangle \in {\text{S}}_{k,l}}{\text{∑}}{\text{r}}_{i,j}^{T}(\text{x})\Lambda_{i,j}{\text{r}}_{i,j}(\text{x})$$

where *k* and *l* denote the indices of the node group and *N_k,l_* is the number of the edges in 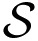
*_k,l_* which represents a set of edges that meets the following conditions.

1. if *k* = *l*: 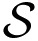
*_k,l_* includes all of the edges that belong to the *k*-th node group, *G_k_*.
2. if |*k* – *l*| = 1: 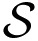
*_k,l_* includes all of the prediction edges between *G_k_* and *G_l_*. The measurement edges that connect *G_k_* and *G_l_* are also included.
3. if |*k* – *l*| > 1: 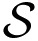
*_k,l_* includes the measurement edges that connect *G_k_* and *G_l_*.

**Figure 12.**
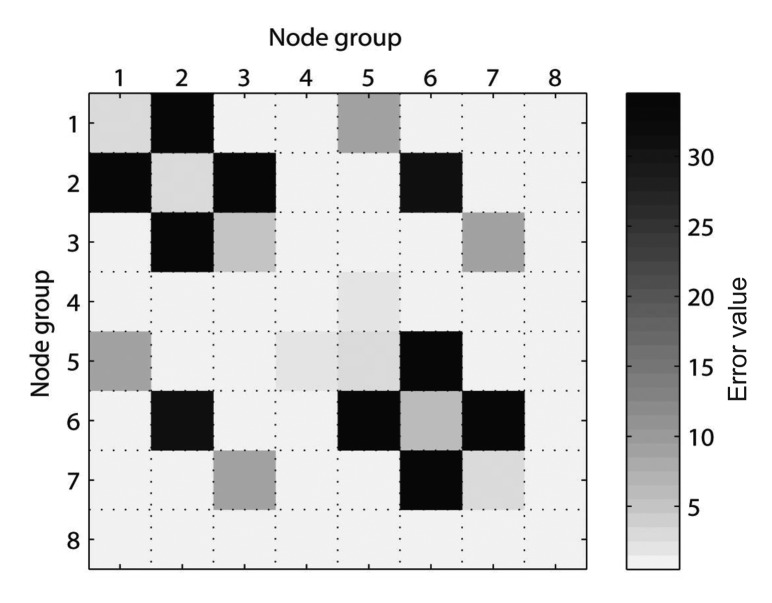
Error metric based on the grouped nodes.

**Figure 13.**
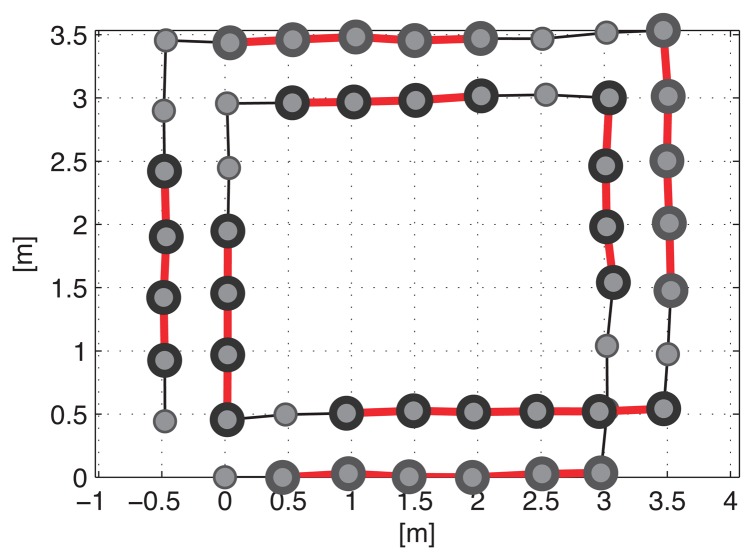
Optimized trajectory after pruning the false constraints.

After applying the error metric to the example distorted pose graph, the error values between the grouped nodes are obtained as shown in Figure 12, in which case the result is represented as symmetric. The information related to the object in *G*_2_ is no longer valid because the object has been moved. Therefore, the false constraints between *G*_2_ and *G*_6_ produce large errors, as indicated by *E*_2,6_. Moreover, the error values *E*_1,2_, *E*_2,3_, *E*_5,6_, and *E*_6,7_ related to the neighbor groups of *G*_2_ and *G*_6_ also produce large values because *G*_2_ and *G*_6_ experience distortion. Finally, considering the error metric results, the error values *E_k,l_* when |*k* – *l*|> 1 are suitable for finding the false edges. Therefore, in this case, according to *E*_2,6_, *G*_2_ that has useless information of the past becomes an object of attention so that the measurement edges related to *G*_2_ are removed. Next, the pose graph is reoptimized and the corrected trajectory of the robot is obtained, as shown in Figure 13. Moreover, *G*_2_ will be excluded from the further process when generating new edges.

This method assumes that inter-group edges are not expected to be large unless landmarks move. However, if there are constraints that were generated by erroneous results of sensors, the edges between inter-group edges would make large error values. Therefore, in this study, we suppose that all erroneous constraints were removed in advance by a variety of robust sensing techniques.

## 4. Experiments

### 4.1. Experimental Setup

**Figure 14.**
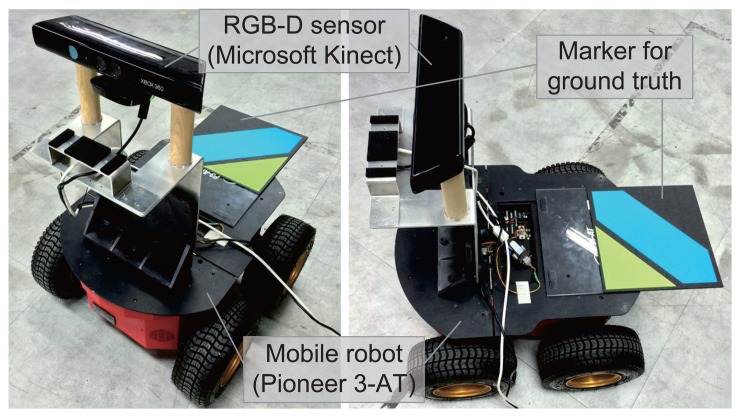
Mobile robot system with an RGB-D sensor and a marker for measuring the ground truth position.

**Figure 15.**
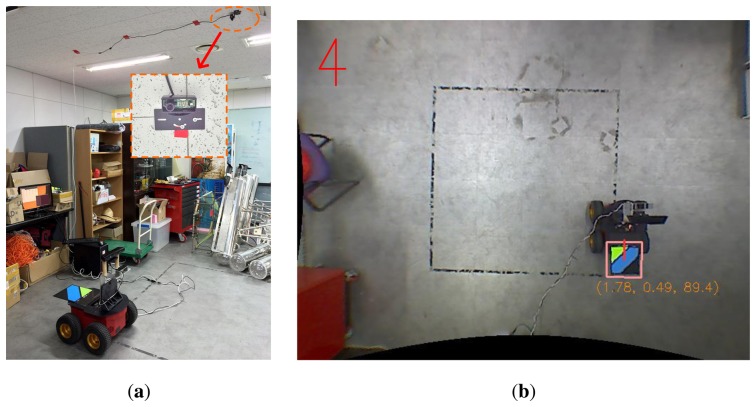
Global vision system for obtaining the ground truth position. (**a**) Camera installed on the ceiling; (**b**) Global positioning result is displayed. A 3-DOF robot pose (*x,y,θ*) is obtained.

**Figure 16.**
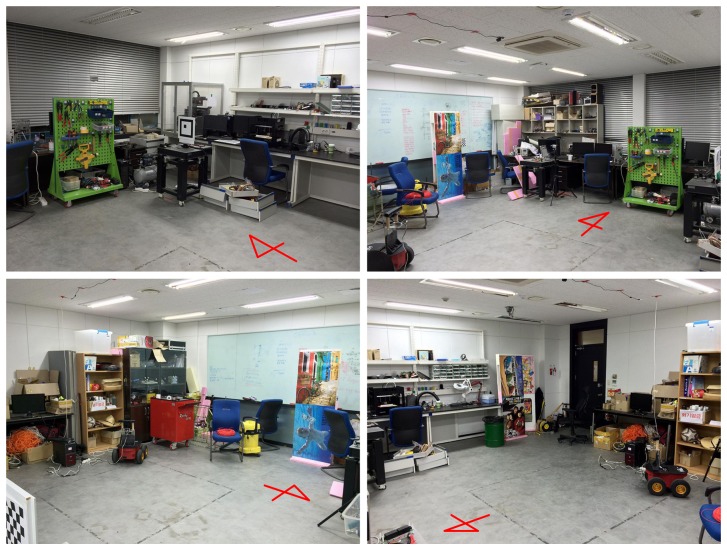
Experimental site used for low dynamic environments.

**Figure 17.**
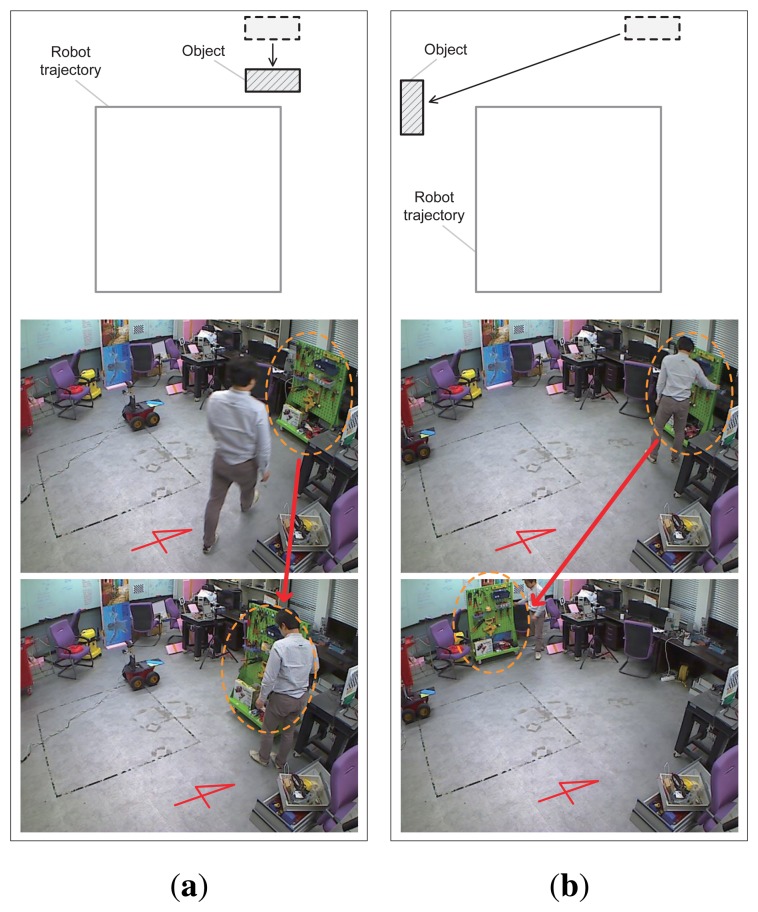
Relocations of the tool cart during two experiments that produce low dynamic environments. (**a**) Experiment 1; (**b**) Experiment 2.

**Figure 18.**
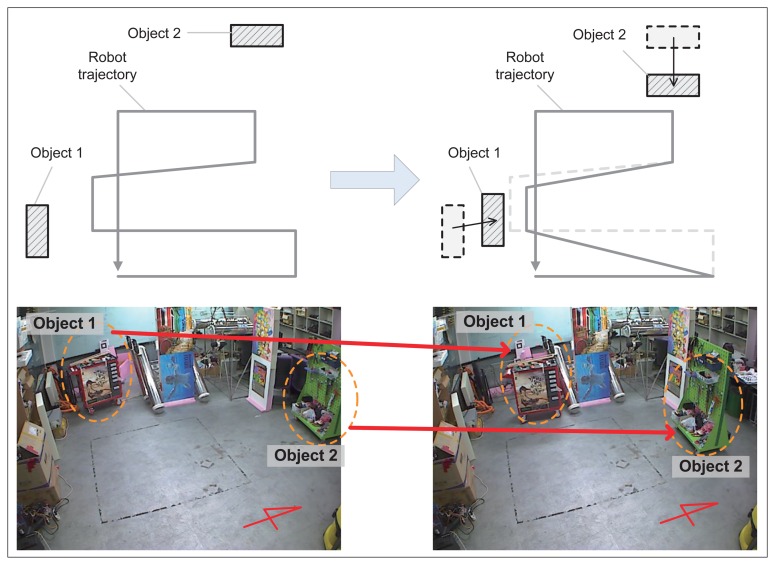
Relocations of the two tool carts during experiment 3 that produces a low dynamic environment.

To validate the proposed method, experiments were performed with a mobile robot. Figure 14 shows the robot system equipped with an RGB-D sensor and a color marker for the ground truth position. The Pioneer 3-AT [24] model was used as the mobile robot where the dead-reckoning data were based only on wheel odometry that was obtained from 100 tick encoders. The RGB-D sensor used in this experiment was Microsoft Kinect [12]. Kinect uses a structured light to estimate depth and its valid range is about 0.5 m to 5 m. The sensor produces a 2D RGB image and per-pixel depth data at 30 Hz, both with 640 × 480 resolution. To measure the ground truth position of the robot, a global vision system was built as shown in Figure 15. A camera was installed on the ceiling and a 3-DOF (degree-of-freedom) robot pose (*x*, *y*, *θ*) was obtained using a marker detection algorithm. The camera covered 4.4 × 3.3 m area, and therefore the resolution of the global vision system was about 0.7 cm per pixel. The experiments were performed in a laboratory environment, as shown in Figure 16. In this setup, we moved tool carts to produce low dynamic environments. Three experiments were conducted using different movements, as shown in Figures 17 and 18. In experiments 1 and 2, the robot moved along rectangular paths, of which the length of one side is 1.8 m, three times. After the first trip, the tool cart was moved to another place. During the second and the third trips, the robot encountered the moved object. In experiment 1, the object was simply moved forward. In experiment 2, the object was relocated to the left-hand side of the experimental setup. Contrary to the experiments 1 and 2 that employed the simple rectangular paths, in experiment 3, a more complex path was built with two moving objects. The left side of Figure 18 shows the first trip. After that, two tool carts were moved to other places, where the robot met during the second trip (the right side of Figure 18). In these settings, the proposed SLAM as well as the conventional RGB-D SLAM were applied.

### 4.2. Experimental Results

**Figure 19.**
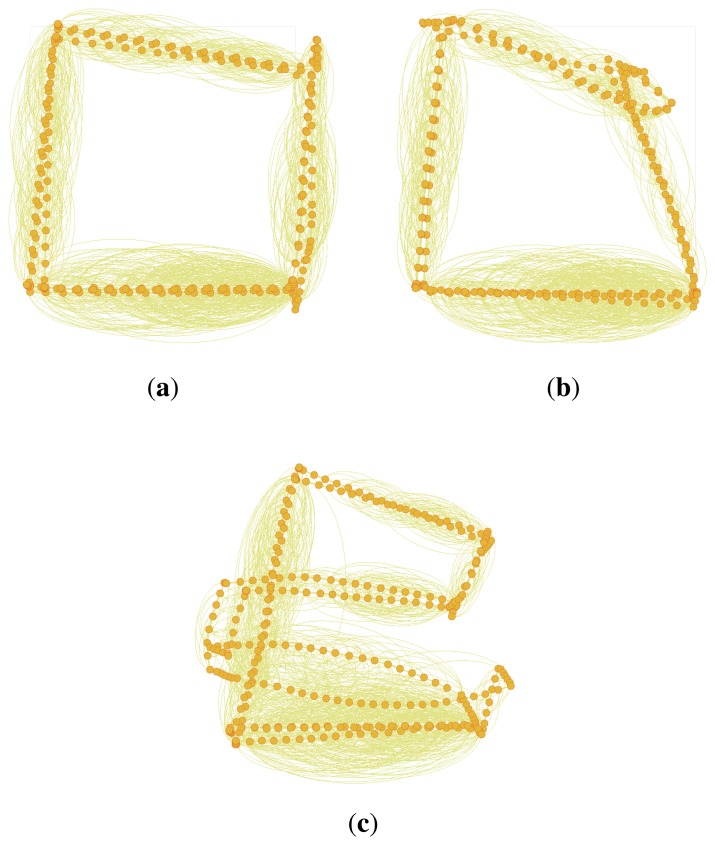
Graph structures obtained from the conventional graph SLAM. The trajectories were distorted due to the low dynamic objects. (**a**) Experiment 1; (**b**) Experiment 2; (**c**) Experiment 3.

**Figure 20.**
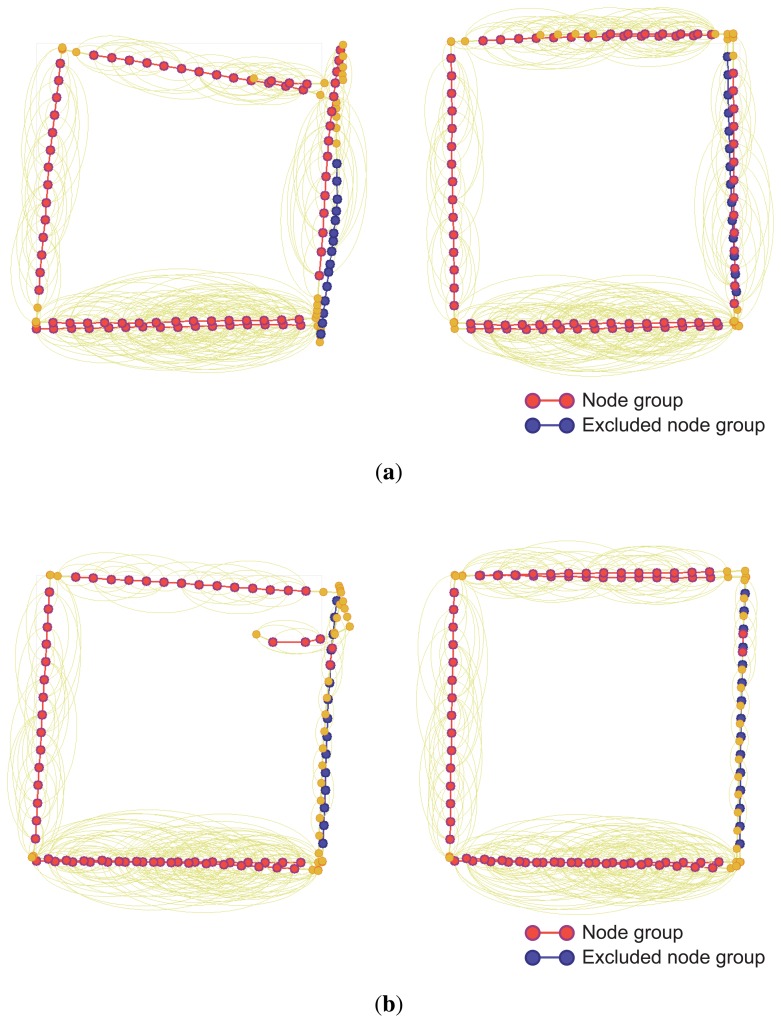
Left: At the moment of movement detection in low dynamic environments. Right: Reoptimized graph structures after excluding false constraints. (**a**) Experiment 1; (**b**) Experiment 2.

**Figure 21.**
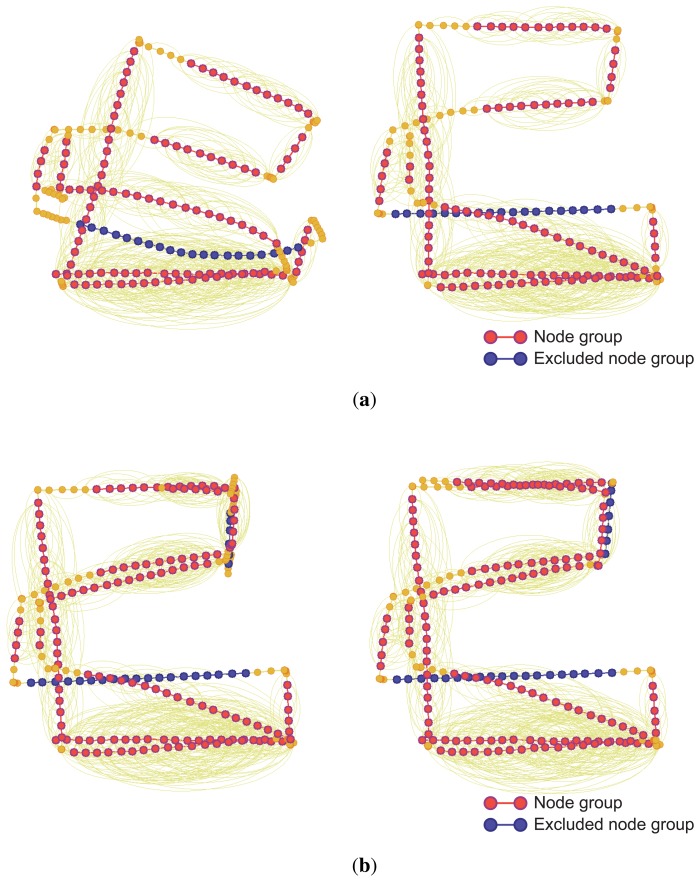
Left: At the moment of movement detection in low dynamic environments. Right: Reoptimized graph structures after excluding false constraints. (**a**) By object 1 in experiment 3; (**b**) By object 2 in experiment 3.

**Figure 22.**
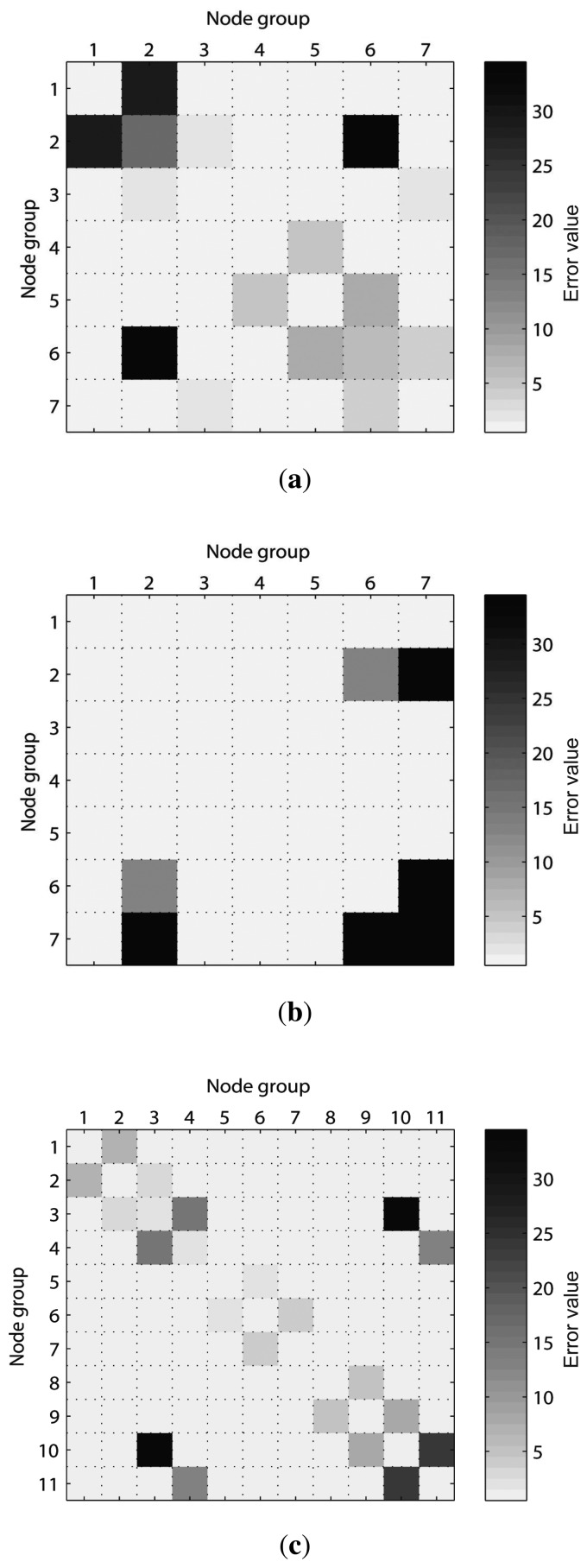

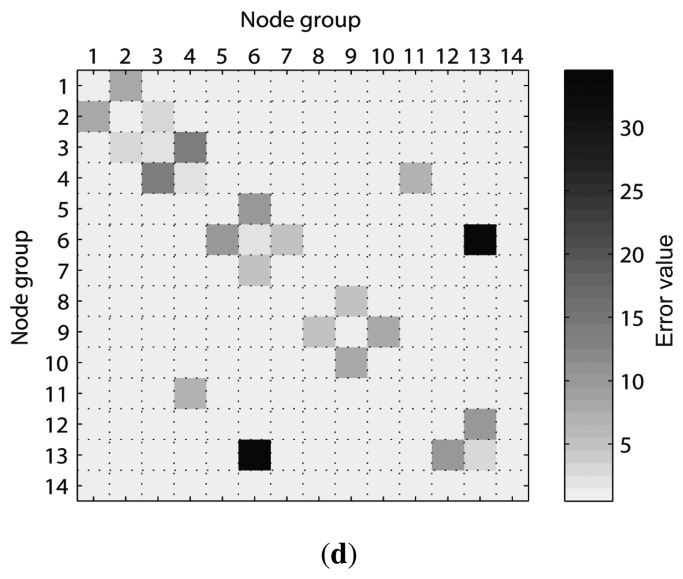
Error metrics for the node groups at the moment of movement detection in low dynamic environments. (**a**) Experiment 1; (**b**) Experiment 2; (**c**) By object 1 in experiment 3; (**d**) By object 2 in experiment 3.

**Figure 23.**
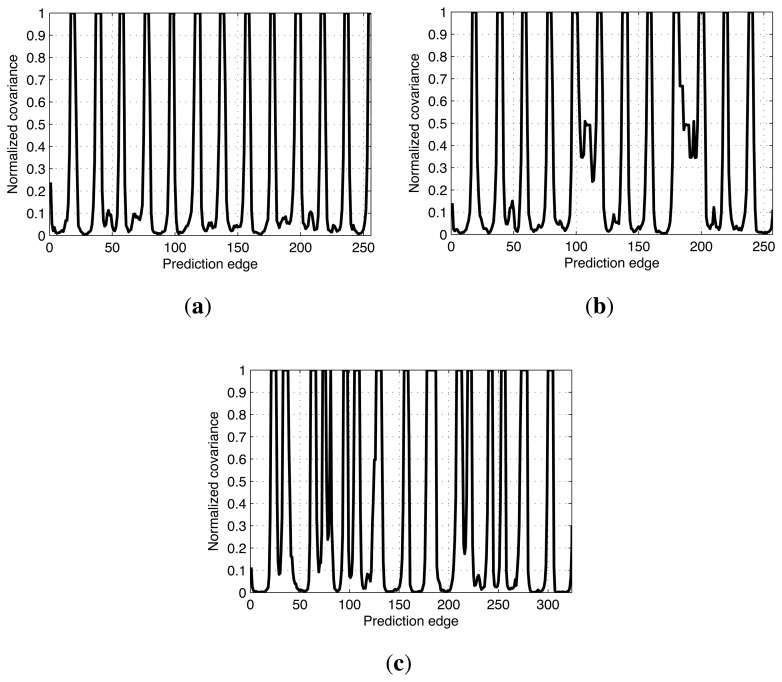
Normalized covariance values of the prediction edges. The nodes in each experiment were grouped based on the covariance values normalized by the maximum value. The nodes with uncertainty values less than 0.3 are grouped. (**a**) Experiment 1; (**b**) Experiment 2; (**c**) Experiment 3.

In the two experiments, the conventional SLAM produced distorted graph structures because of the low dynamic objects, as shown in Figure 19. However, the proposed method detected the dynamic environments using the error metric, as shown in Figures 20–22. At this time, the nodes were grouped based on the covariance values which were normalized by the maximum value, as shown in Figure 23. The nodes with uncertainty values less than 0.3 are grouped. In Figures 20 the left column shows the moment of detection at the second turn in experiments 1 and 2. At these times, the false constraints were excluded based on the error metrics of the node groups in experiments 1 and 2 (Figure 22a,b).

In experiment 1, the error value *E*_2,6_ had a high score and *G*_2_ was excluded. In the same manner, *E*_2,7_ had a large error value in experiment 2 and the edges connected to *G*_2_ were removed. In Figures 21, the left column shows the moment of movement detection by object 1 and 2 at the second turn in experiments 3. The false constraints related to *G*_3_ and *G*_6_ also were excluded based on the error metrics (Figures 22c,d). After pruning the false constraints, the modified graph structures were optimized by the graph SLAM algorithm from the beginning. Although the reoptimization started through the process all over again, the iSAM algorithm provided sufficient performance for real-time operation. The reoptimized graph structures are shown in the right column of Figures 20 and 21.

**Figure 24.**
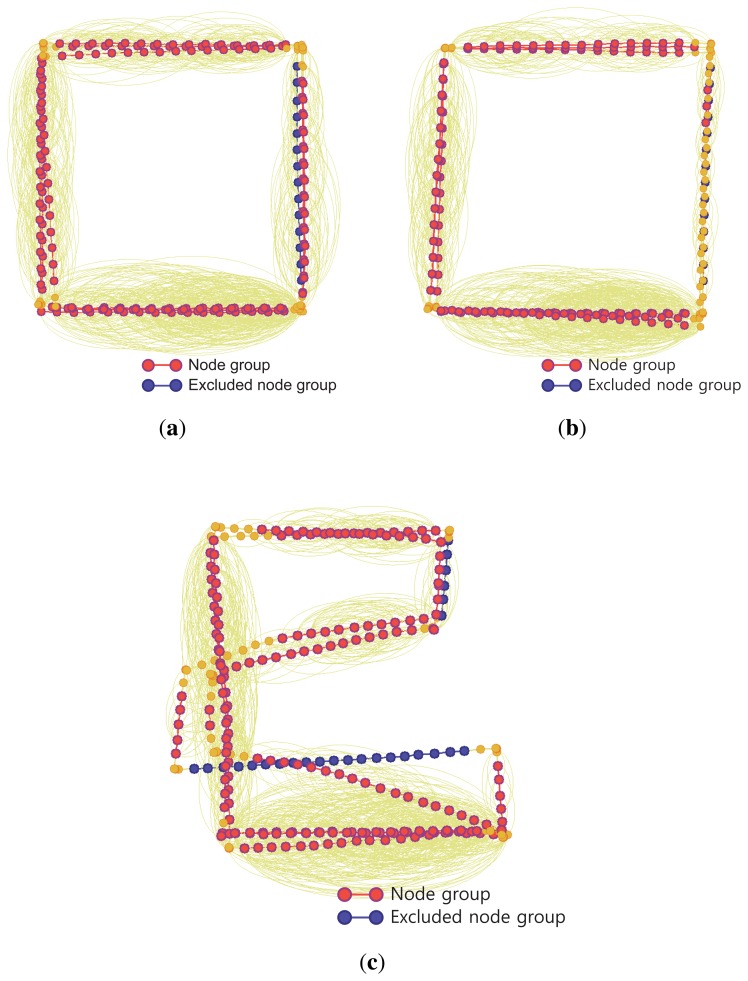
Reoptimized graph structures obtained using the proposed method. (**a**) Experiment 1; (**b**) Experiment 2; (**c**) Experiment 3.

**Figure 25.**
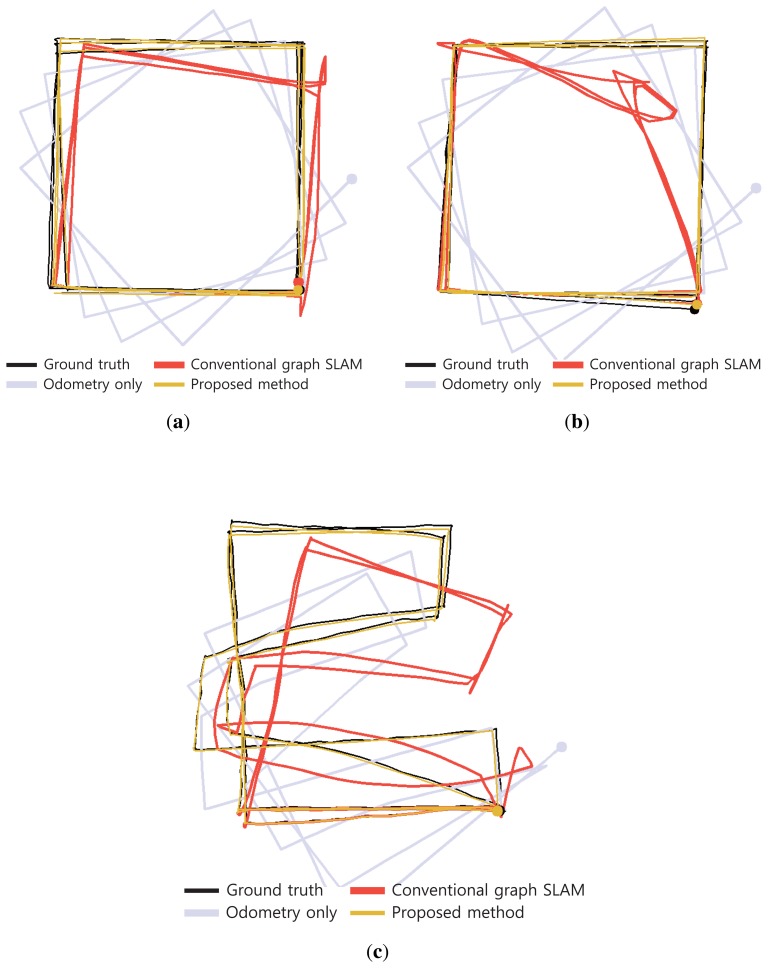
Four types of robot trajectories obtained in low dynamic experiments. (**a**) Experiment 1; (**b**) Experiment 2; (**c**) Experiment 3.

The final results for the overall trajectories are shown in Figures 24 and 25. The graph structures obtained from SLAM are shown in Figure 24. The node group *G*_2_ is excluded from experiment 1 and 2. In experiment 3, two node groups *G*_3_ and *G*_6_ are excluded due to the movements of the two objects. In Figure 25, three types of trajectories are compared with the ground truth result for each experiment. The results obtained using odometry deviate increasingly from the ground truth because of wheel odometry errors. The conventional graph SLAM results show distorted trajectories due to low dynamic objects. However, the results obtained using the proposed method agree well with the ground truth.

**Figure 26.**
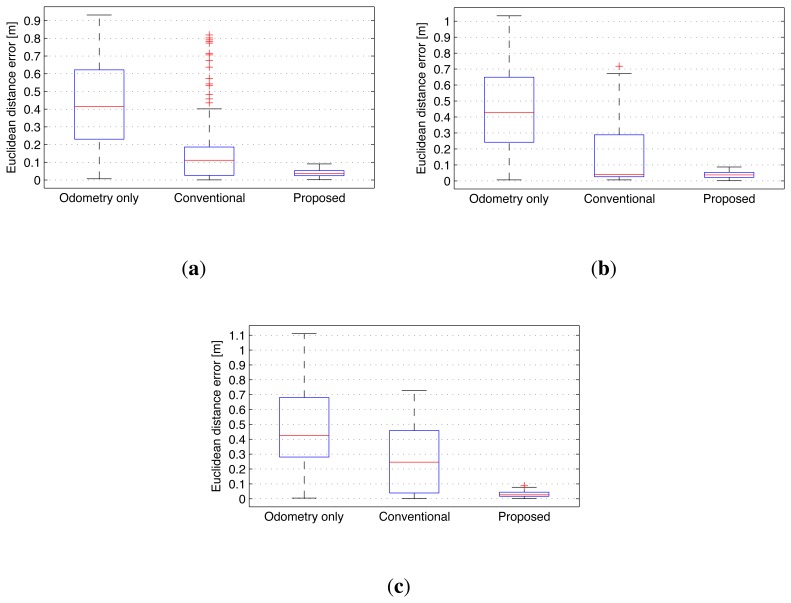
Euclidean distance errors relative to the ground truth data. (**a**) Experiment 1; (**b**) Experiment 2; (**c**) Experiment 3.

The Euclidean distance errors of the three results were calculated relative to the ground truth data and they are shown as boxplots in Figure 26. The median values of the odometry only results were 0.415 m, 0.429 m, and 0.424 m in experiments 1, 2, and 3, respectively. The median values with the conventional SLAM results were 0.112 m, 0.041 m, and 0.246 m in experiments 1, 2, and 3, respectively. With the proposed method, the median values were 0.037 m, 0.038 m, and 0.026 m in experiments 1, 2, and 3, respectively. Thus, in experiment 2, the median values obtained with conventional SLAM and the proposed method did not differ greatly. However, the conventional SLAM had a large variance (the third quartile was 0.289 m).

**Figure 27.**
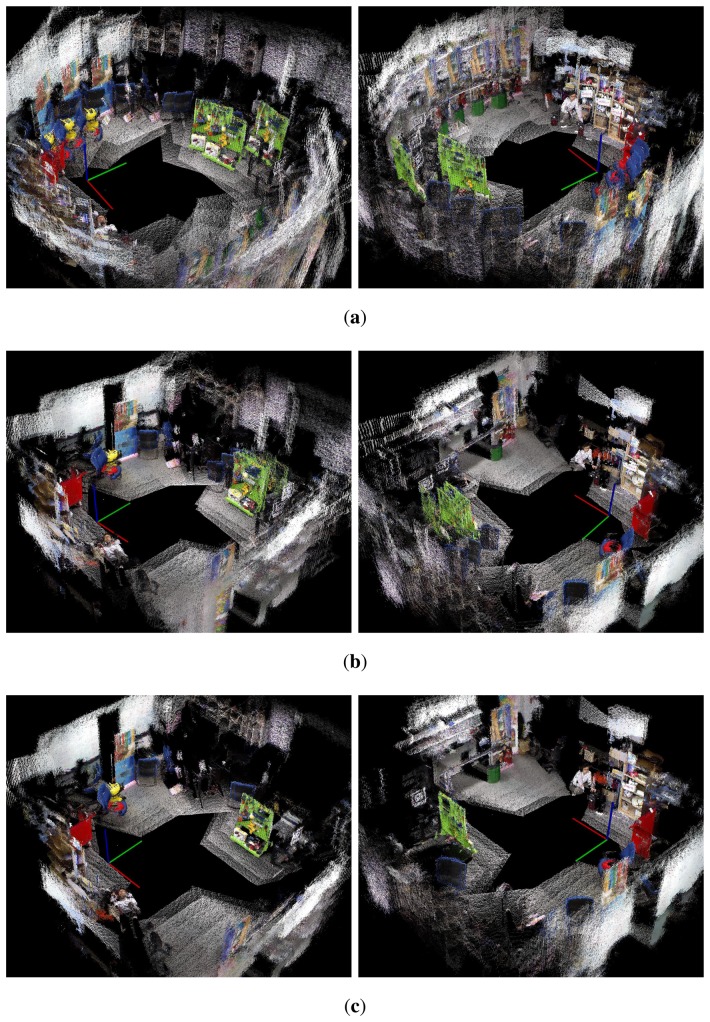
The 3D maps obtained from experiment 1. Left and right columns are views from different angles. (**a**) Odometry only; (**b**) Conventional graph SLAM; (**c**) Proposed method.

**Figure 28.**
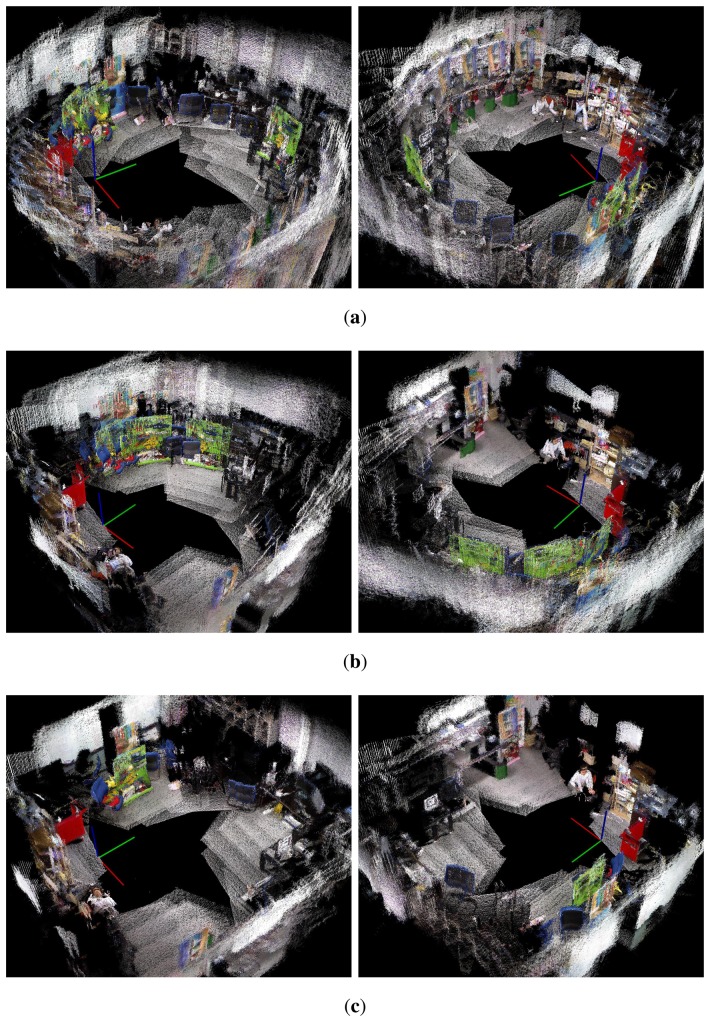
The 3D maps obtained from experiment 2. Left and right columns are views from different angles. (**a**) Odometry only; (**b**) Conventional graph SLAM; (**c**) Proposed method.

**Figure 29.**
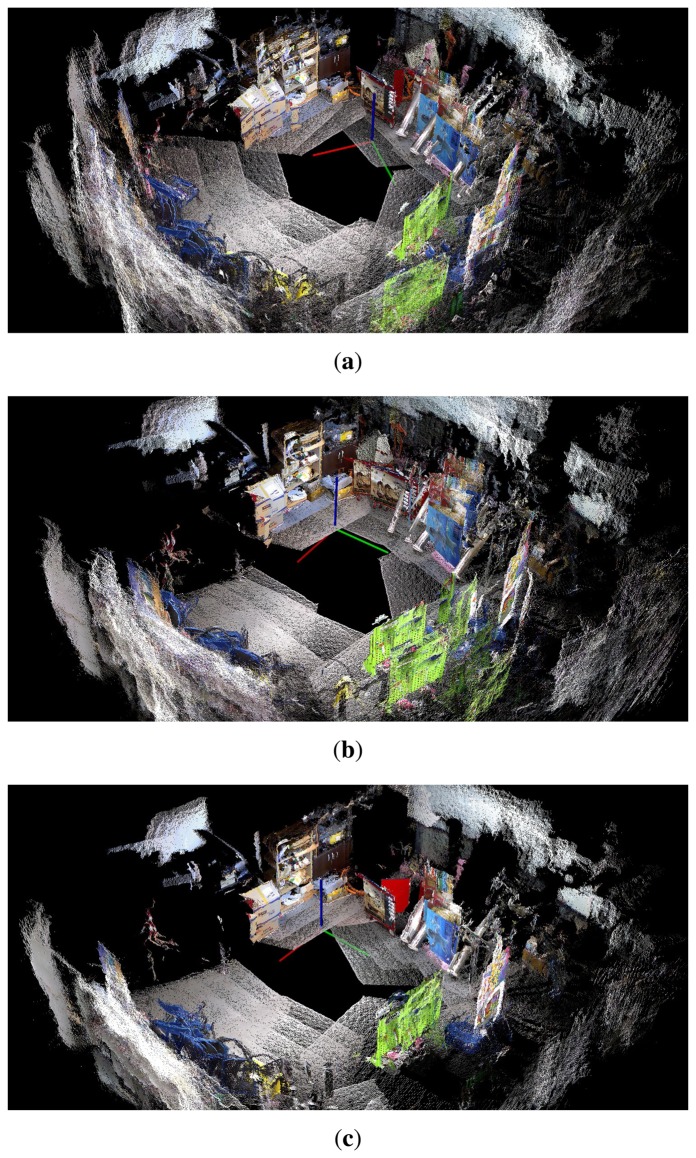
The 3D maps obtained from experiment 3. (**a**) Odometry only; (**b**) Conventional graph SLAM; (**c**) Proposed method.

Figures 27–29 show the 3D maps obtained from the experiments. The 3D map was constructed by merging the point cloud data from all the nodes in the graph structures. In Figures 27a, 28a, and 29, it is difficult to recognize the experimental site because of the odometry errors. The results obtained with conventional graph SLAM (Figures 27b, 28b, and 29b) show that the tool cart appears in several positions. In experiment 2, the tool cart traveled further than in experiment 1 and the error in the 3D map was more significant. In Figures 27c, 28c, and 29c, however, the proposed method produced the correct 3D maps using the reoptimized graph and by removing the point cloud data for the excluded nodes. Therefore, the traces of the object before moving could be removed completely.

## 5. Conclusions

In this study, we proposed an RGB-D SLAM method that handles low dynamic situations using a pose-graph structure. Nodes that observe the same object using a sensor are grouped based on their covariance values. Any false constraints are pruned based on an error metric related to the node groups. The validity of the proposed method was demonstrated by real experiments in low dynamic environments. The corrected trajectories of a robot and 3D maps that contained the final appearance of the dynamic object were obtained successfully.

It is expected that this method will help to improve the performance of graph SLAM in various dynamic environments. In the present study, the robot pose movements were limited to the 2D plane. Therefore, further studies should be conducted using 6-DOF movements in 3D spaces.
